# Synthesis of nitrogen-doped graphene wrapped SnO_2_ hollow spheres as high-performance microwave absorbers

**DOI:** 10.1039/c9ra01556f

**Published:** 2019-04-05

**Authors:** Weiyong Dai, Hui Luo, Fu Chen, Xian Wang, Ying Xiong, Yongzhi Cheng, Rongzhou Gong

**Affiliations:** School of Optical and Electronic Information, Huazhong University of Science and Technology Wuhan 430074 People's Republic of China; School of Information Science and Engineering, Wuhan University of Science and Technology Wuhan 430081 People's Republic of China luohui@wust.edu.cn

## Abstract

Nitrogen-doped graphene (NG)/SnO_2_ hollow sphere hybrids were synthesized in this work. The chemical composition, crystal structure and morphology have been characterized by FT-IR spectra, XRD, Raman spectra, XPS, SEM and TEM in detail. Reflection loss (RL) values of NG/SnO_2_ hollow sphere hybrids less than −10 dB and −20 dB are found in the wide frequency range of 4.5–18 GHz and 5–16.2 GHz within 1.3–3.5 mm, and a minimum RL of −50.3 dB is achieved at 8.6 GHz with the matching thickness of only 2.3 mm. The results indicate that the NG/SnO_2_ hollow sphere hybrids with high-performance microwave absorption properties have a promising future in decreasing electromagnetic wave irradiation and interference.

## Abstract 2

Nitrogen-doped graphene/SnO_2_ hollow sphere hybrids were synthesized and show high-performance microwave absorption properties.

## Graphical abstract

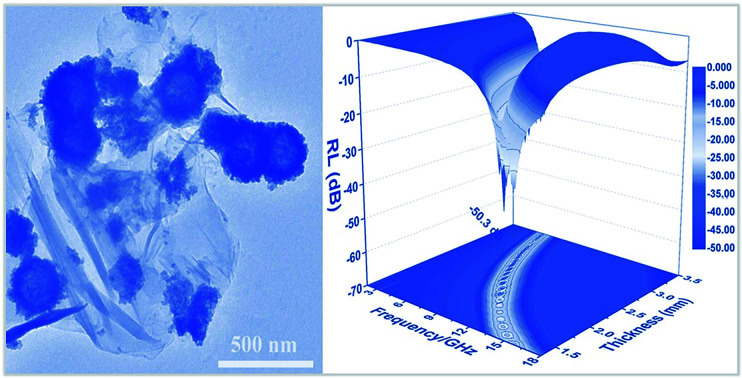

## 1. Introduction

Electromagnetic (EM) wave absorbing materials with the properties of broad bandwidth, strong absorption, light weight and thin-thickness have attracted much attention due to increasing EM interference and radiation.^1–7^ The EM absorption properties of an absorber are affected by complex permittivity, complex permeability, EM impedance match and microstructure.^1,8^ Carbon materials, such as carbon black, graphene and carbon nanotubes have been used as microwave absorbing materials in recent years for their light weight, low cost and highly electrically conductive properties.^9–14^ Nevertheless, graphene has a poor on/off current ration due to its zero-band-gap, which is not good for EM wave absorption.^15^ Doping atoms such as N and S into the graphene lattice to form covalent bonds with neighboring carbon atoms, leads to the modification of the electronic structure of graphene and may generate more defects, which can act as polarization and scattering centers and can be beneficial for microwave absorption.^15–18^ The graphene has high dielectric loss properties but exhibited low EM absorption property due to its poor impedance matching. It had been found that coupling nanostructured materials with graphene sheets could improve the EM wave absorption properties significantly in recent studies.^19–28^ For graphene-based composites, the real part of permittivity exhibits a sharp decrease, while the imaginary part shows only a slight decrease compared with graphene.^19^ The combination of a binary dielectric or magnetic material with graphene not only benefits its attenuation ability, but also obtain a good impedance matching.^20–28^

Hollow structures exhibit special physical and chemical properties due to their low density and large surface area, having attracted attention as candidates for microwave absorption in recent years.^29–31^ Zhao *et al.* prepared the hollow three-dimensional CuS hierarchical microspheres and the effective bandwidth was 3.0 GHz with absorber thickness of 1.1 mm.^32^ Considering the outstanding properties of graphene as well as hollow structures, hollow structures/graphene hybrids would be very attractive for practical applications. Han *et al.* synthesized the graphene-wrapped ZnO hollow spheres and a maximum absorption of −45.05 dB at 9.7 GHz with the thickness of 2.2 mm.^33^ Fu *et al.* prepared CoFe_2_O_4_ hollow spheres/graphene composites and a minimum reflection loss of −18.5 dB was observed at 12.9 GHz with a thickness of 2 mm and the effective absorption frequency range from 11.3 to 15.0 GHz.^34^

SnO_2_ as one of the most important semi-conductor transition-metal (band gap 3.6 eV) with temperature and environment stable dielectric properties, has been studied intensively by some groups acting as microwave absorbing materials.^35–41^ Zhao *et al.* synthesized the order honeycomb-like SnO_2_ foams and reflection loss values less than −10 dB reaches 5.6 GHz with a thin thickness of 2.0 mm.^36^ The SnO_2_ act as shells of Fe_3_O_4_, Ni, Fe *etc.* also have been studied and obtained good microwave absorption properties.^37–39^ The composites with SnO_2_ decorated on graphene sheets have been used for supercapacitor application and microbial fuel cells.^42,43^ So far, the EM absorption properties of N-doped graphene/SnO_2_ hollow spheres hybrids have not been studied.

Herein, NG/SnO_2_ hollow spheres hybrids were fabricated using a hydrothermal method with SiO_2_ act as sacrificial template, urea act as nitrogen source and reducing agent in this work. The microwave absorption performance of NG/SnO_2_ hollow spheres hybrids was investigated in the frequency range of 2–18 GHz. The high performance EM absorbing mechanism of the NG/SnO_2_ hollow spheres hybrids is further discussed.

## 2. Experimental section

All the chemicals and reagents were used without further purification. Graphene oxide (GO) was synthesized from natural graphite by using a modified Hummers' method.^44^ The silica nanospheres with particle size of about 200 nm were prepared *via* a Stöber method.^45^

### 2.1. Preparation of SnO_2_ hollow spheres

The SnO_2_ hollow spheres were prepared by according to previous method with some modification.^45,46^ Typically, 100 mg of as-prepared SiO_2_ nanospheres were dispersed into 60 mL of deionized water and ultrasound for 2 h. Then, 1.2 g of glucose was added into the suspension and stirred for 10 min. After that, 0.2 g SnCl_2_·2H_2_O was added into the suspension under stirring. After stirring for 30 min, 0.044 g of NH_4_F was added into the mixed suspension and stirred another 30 min. After that, the mixed suspension was transferred into a Teflon-lined stainless steel autoclave, and then placed in an oil bath at 160 °C with magnetic stirring for 10 h. After cooled down to room temperature, the hollow SnO_2_@polysaccharides nanospheres were collected by centrifugation, washed with deionized water and ethanol thoroughly, and dried in an oven at 60 °C. Finally, the SnO_2_ hollow spheres composite was obtained by heat treatment at a temperature of 500 °C for 3 h with a ramping rate of 4 °C min^−1^ in air.

### 2.2. Preparation of NG/SnO_2_ hollow spheres hybrids

Amino-function SnO_2_ hollow spheres (amino-SnO_2_) were achieved *via* a post-grafting strategy. Typically, 100 mL of anhydrous toluene and 200 mg of SnO_2_ hollow spheres was mixed under ultrasound and mechanical stirring for 1 h. Then 0.5 mL of APTES was added dropwise to the suspension and kept at 110 °C with reflux for 6 h. The amino-SnO_2_ were collected by centrifugation and washed for several times with ethanol and deionized water and dried in an oven at 60 °C. The NG/SnO_2_ hollow spheres hybrids were prepared by using a hydrothermal method. Briefly, 100 mg GO was dispersed into 60 mL deionized water and ultrasound for 2 h, 100 mg amino-SnO_2_ hollow spheres were added into the suspension with ultrasonic and stirred for 1 h. Then 2 g urea was added into the suspension and stirred for another 30 min. The suspension was transferred into a stainless-steel autoclave and kept at 180 °C for 6 h. The product was collected by centrifugation and then washed with ethanol and deionized water and dried at 60 °C. The NG was also prepared by using the same method without amino-SnO_2_.

### 2.3. Characterization

The crystal phase was investigated with X-ray powder diffraction (Empyrean, PANalytical B.V.). Raman spectroscopy was carried out on a LabRAM HR800 in *via* Raman microscope. X-ray photoelectron spectroscopy (XPS) measurements were obtained with an AXIS-ULTRA DLD-600W electron spectrometer from Kratos. The morphology was examined by field emission scanning electron microscope (Gemini SEM 300) and transmission electron microscope (TEM, Tecnai G2 20). The measurements of FT-IR were performed with spectroscopy (VERTEX 70) using the KBr pellet method at room temperature. The electromagnetic parameters of the samples were measured by a vector network analyzer (Agilent E5071C) within 2–18 GHz. The toroidal shape samples with an inner diameter of 3.04 mm, out diameter of 7 mm and thickness of 2 mm were prepared by mixing the paraffin and samples with a mass ratio of 6 : 4.

## 3. Results and discussion

**Fig. 1:**
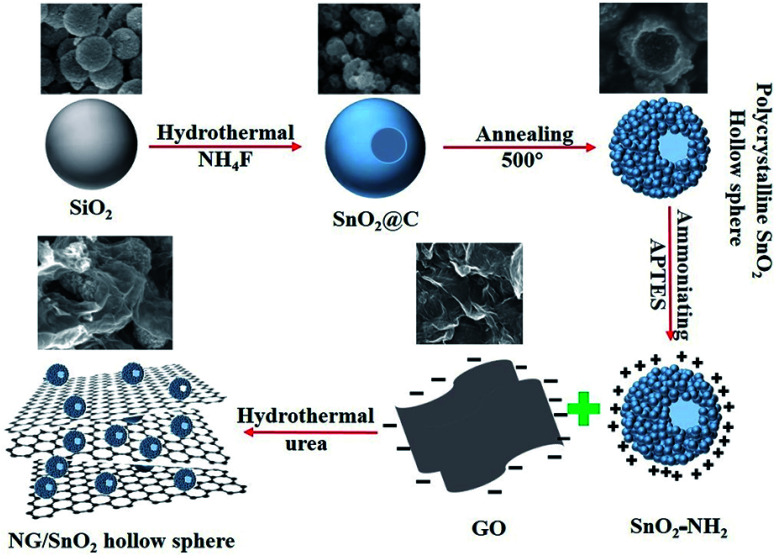
Schematic illustration for the formation mechanism of NG/SnO_2_ hollow spheres hybrids.

The formation mechanism of NG/SnO_2_ hollow spheres hybrids is schematically illustrated in Fig. 1. First, polycrystalline SnO_2_ hollow spheres were prepared by facile strategy of one-pot hydrothermal following a subsequent heat treatment with SiO_2_ act as sacrificial template. Second, amino-functional SnO_2_ hollow spheres were obtained *via* a post-grafting strategy with reflux. Third, amino-function SnO_2_ hollow spheres anchored on the GO sheets surface through electrostatic interaction and the NG/SnO_2_ hollow spheres hybrids were prepared by using a hydrothermal method with urea as the nitrogen source and reducing agent.

**Fig. 2:**
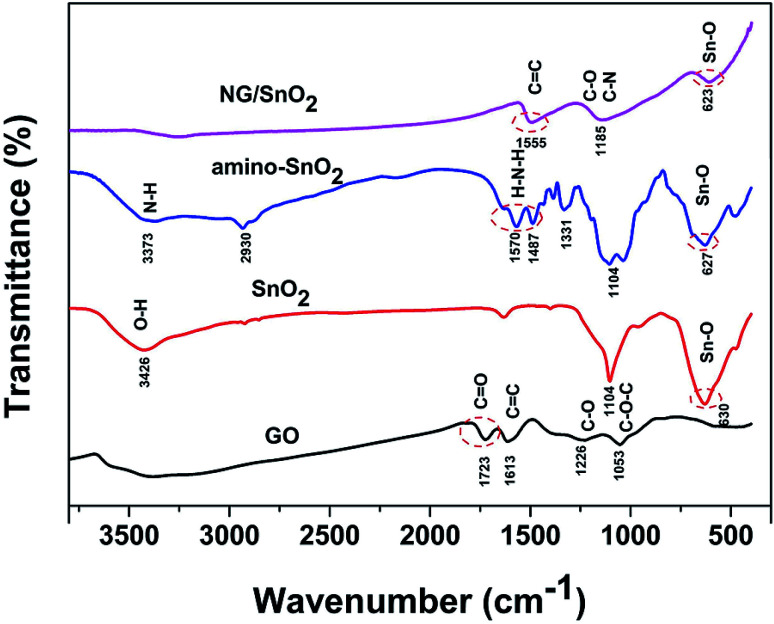
The FT-IR spectra of GO, SnO_2_, amino-SnO_2_ and NG/SnO_2_ hollow spheres hybrids.

Fig. 2 shows the FT-IR spectra of the samples at each stages of the experiments. The characteristic features of GO are the adsorption bands corresponding to the C=O stretching vibration at 1723 cm^−1^, C=C deformation vibration at 1613 cm^−1^, C–OH stretching at 1226 cm^−1^ and C–O stretching at 1053 cm^−1^, indicating that abundant oxygen functional groups (–COOH, –OH) were introduced during the exfoliation of the graphene sheet.^2^ Compared the FT-IR spectrum of amino-function SnO_2_ hollow spheres with SnO_2_ hollow spheres, two new bonds were found at 3373 and 1570 cm^−1^ correspond to the coupling of N–H stretching vibration, suggesting that NH_2_ group have adhered to the surface of SnO_2_ hollow spheres.^47^ All samples except for GO show strong absorption at 500–700 cm^−1^ corresponding to characteristic vibration of O–Sn–O in the SnO_2_.^48^ For NG/SnO_2_ hollow spheres hybrids, the peaks at 1723, 1226 and 1503 cm^−1^ almost disappear, indicating that most oxygen function groups have been removed.^2^ There are three peaks at 1555, 1185 and 623 cm^−1^ can be assigned to the C=C, C–N(C–O) and Sn–O stretching vibration in NG/SnO_2_ hollow spheres hybrids, indicating that the SnO_2_ hollow spheres have been successful anchored on the NG surface.

**Fig. 3:**
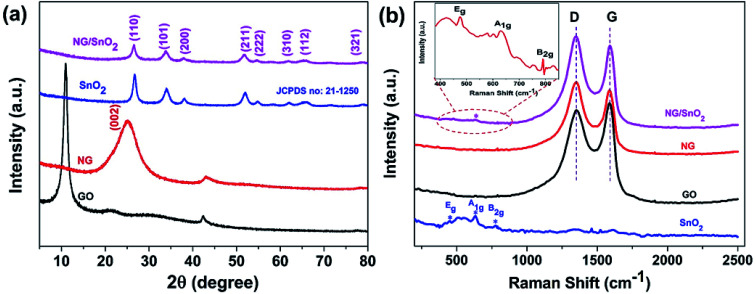
XRD patterns (a) and Raman spectra (b) of the GO, NG, SnO_2_ hollow spheres and NG/SnO_2_ hollow spheres hybrids.

The XRD was utilized to investigate the phase structure of GO, NG, SnO_2_ hollow spheres and NG/SnO_2_ hollow spheres hybrids, as shown in Fig. 3(a). The GO shows a sharp peak at 10.9°, corresponding to its (001) crystal plane. The NG shows one significant diffraction peaks at 2*θ* = 25.3° attributed to the (002) reflection of graphitic carbon.^49^ The XRD pattern with the (110), (101), (200), (211), (222), (310), (112), and (321) planes confirmed the formation of SnO_2_ (JCPDS no. 21-1250). No apparent diffraction peak could be found at around 25° for NG/SnO_2_ hollow spheres hybrids, indicating that SnO_2_ hollow spheres have been decorated on the NG sheets surface, suppressing the stacking of graphene layers. Fig. 3(b) shows the Raman spectra of the samples, there are three characteristic peaks for SnO_2_ at 472, 632 and 775 cm^−1^ corresponding to E_g_, A_1g_ and B_2g_ modes of crystalline SnO_2_, respectively.^50^ For GO, NG and NG/SnO_2_ hollow spheres hybrids, two apparent peaks centered at 1350 and 1590 cm^−1^ are attributed to the D bands associated with structure defects and G bands for the E_2g_ vibration mode of sp^2^ carbon domains of carbon materials, respectively. The ratio of the intensity of D-band to G-band (*I*_D_/*I*_G_) is a measure of the degree of disorder in the graphene or GO.^51^ The D and G bands cannot be found in SnO_2_ hollow spheres, indicating that there is no carbon on the surface of SnO_2_ hollow spheres. There are three additional peaks corresponding to the SnO_2_ in the NG/SnO_2_ hollow spheres hybrids indicating that the SnO_2_ hollow spheres were efficiently deposited on the NG surface. The *I*_D_/*I*_G_ increases from 0.97 for GO to 1.1 for NG/SnO_2_ hollow spheres hybrids, which suggest that more defects are formed during the SnO_2_ hollow spheres anchored on the NG surface and the reduction of GO.^52^ Both XRD and Raman measurement confirmed the successful integration of NG and SnO_2_ hollow spheres.

**Fig. 4:**
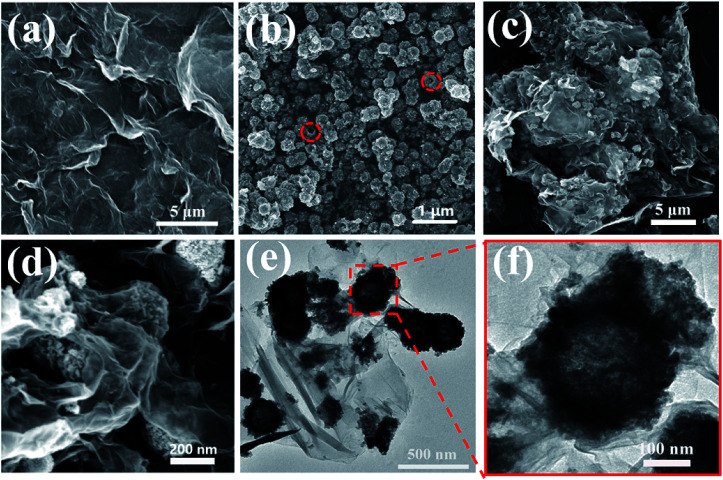
SEM images of GO (a), SnO_2_ hollow spheres (b), and NG/SnO_2_ hollow spheres hybrids (c) and (d), TEM imagine of NG/SnO_2_ hollow spheres hybrids (e) and (f).

The morphologies and structures of GO, SnO_2_ hollow spheres and NG/SnO_2_ hollow spheres hybrids were further investigated by FESEM and TEM. As showed in Fig. 4(a), the aggregated GO powder shows a flaky crumple wrinkles.^1^ High resolution SEM image clearly shows that the SnO_2_ hollow spheres with an average diameter of 220 nm, as displayed in Fig. 4(b). From Fig. 4(c) and (d), it can be obviously seen that the SnO_2_ hollow spheres are uniformly anchored on the NG surface, forming sandwich-structure NG/SnO_2_ hollow spheres hybrids. Fig. 4(e) and (f) reveals the TEM images of NG/SnO_2_ hollow spheres hybrids, the NG is nearly transparent with lots of wrinkles on its surface and the hollow feature of SnO_2_ nanospheres is further confirmed by TEM image (Fig. 4(f)).^1^

**Fig. 5:**
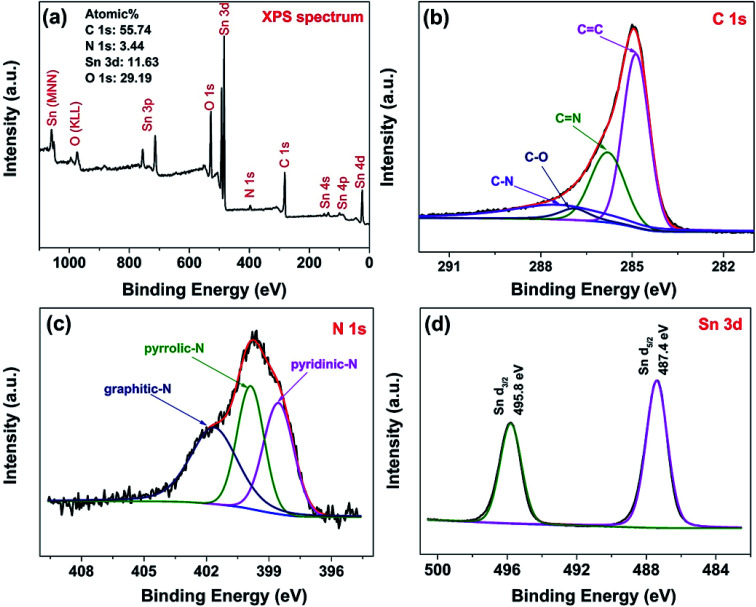
Survey scan (a), C 1s spectrum (b), N 1s spectrum (c) and Sn 3d spectrum (d) of NG/SnO_2_ hollow spheres hybrids.

The surface chemical composition of the hybrids were characterized by XPS technology. Fig. 5(a) shows the survey scan XPS spectrum of NG/SnO_2_ hollow spheres hybrids which shows that the hybrids consist of Sn, O, C and N elements. The atomic percentage of N in the sample is about 3.44%, indicating that some C atom was replaced by N atom. From the high resolution spectrum of C 1s, the main peak at 284.9 eV corresponds to the graphite-like sp^2^ C (C=C), indicating most of the C atoms in the N-doped graphene are arranged in a conjugated honeycomb lattice. The peaks at 285.8 and 287.5 eV reflect different bonding structure of C–N bonds, N-sp^2^ C(–C=N) and N-sp^3^ C(–C–N) bonds, respectively, which originate from substitution of N atom, defects or the edge of the graphene sheets.^53^ Small peaks at 286.9 eV reflect different bonding structure of C–O bonds, indicating that there are also some oxygen functional groups on NG surface. Deconvolution of the high-resolution N 1s spectra shows distinct N-containing functional group coexisted, identified by their unique bonding states in the hybrids.^54^ The peaks locate at 398.6, 400 and 401.7 eV can be attributed to pyridinic-N, pyrrolic-N and graphitic-N respectively.^54^ Two symmetrical peaks at 487.4 eV and 495.8 eV are attributable to Sn 3d_5/2_ and Sn 3d_3/2_ are showed in Fig. 5(d), respectively, which is in good agreement with the energy splitting reported for SnO_2_.^55^

**Fig. 6:**
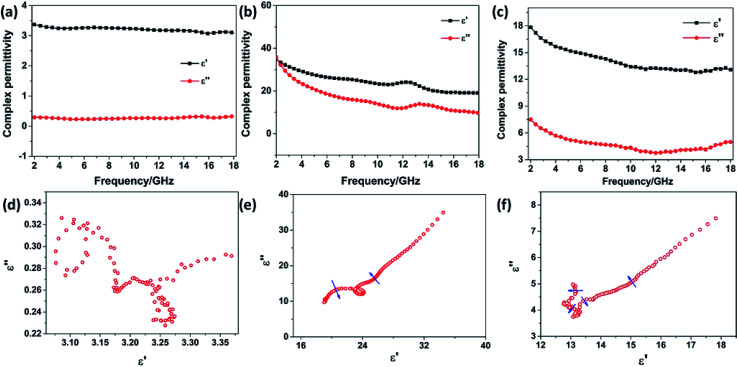
Complex permittivity *versus* frequency of SnO_2_ hollow spheres (a), NG (b) and NG/SnO_2_ hollow spheres hybrids (c), the Cole–Cole semicircles for SnO_2_ (d), NG (e) and the NG/SnO_2_ hollow spheres hybrids (f).

The frequency dependence of the complex permittivity and complex permeability for the SnO_2_ hollow spheres, NG and NG/SnO_2_ hollow spheres hybrids were are shown in Fig. 6. The real part (*μ*′) and imaginary part (*μ*′′) of the complex permeability of the samples are close to 1 and 0, respectively, due to the SnO_2_ and NG is nonmagnetic at room-temperature. The real part (*ε*′) and imaginary part (*ε*′′) for SnO_2_ hollow spheres are nearly constant within 2–18 GHz, with an inconspicuous undulation (*ε*′ ≈ 3.2 and *ε*′′ ≈ 0.28) as shown in Fig. 6(a). It can be observed that the *ε*′ and *ε*′′ for NG decrease gradually from 34.51 to 19.03 and 34.93 to 9.69, respectively, with a resonance peak at about 13.5 GHz. Fig. 6(c) shows the relative complex permittivity of NG/SnO_2_ hollow spheres hybrids. The *ε*′ values gradually decrease from 17.8 to 12.7 with the increase of the frequency and the *ε*′′ values are varied in the range of 3.78–7.5 over 2–18 GHz. The complex permittivity of NG/SnO_2_ hollow spheres hybrids is enhanced in the whole frequency range after the NG introduced compared with SnO_2_ hollow spheres, which should be ascribed to the increased electrical conductivity in the presence of NG. The obvious improvement of complex permittivity for NG/SnO_2_ hollow spheres hybrids is possibly due to relaxation polarization of the residual defect and groups of the NG surface which are generated by the reduction and N doping process of GO during the formation of NG/SnO_2_ hollow spheres hybrids.

On the basis of Debye relaxation, the relative complex permittivity can be expressed by the following equation:^13,36^1
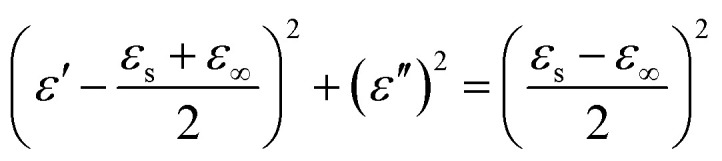
where *ε*_s_ and *ε*_∞_ are the static dielectric constant and the dielectric constant at infinite frequency, respectively.^2^ It is clear that the plot of *ε*′ *versus ε*′′ would be a single semicircle, which is denoted as a Cole–Cole semicircle. Each semicircle is linked to one Debye relaxation process.^1,2^Fig. 6(e) shows the plots of *ε*′′ *versus ε*′ for NG and present two semicircles corresponding to two relaxation processes. Nevertheless, four semicircles are obviously obtained in the curve of the NG/SnO_2_ hollow spheres hybrids (Fig. 6(f)) corresponding to four Debye relaxation processes, which suggesting there are multiple mechanisms such as interfacial polarization and dipole orientation polarization. The interfacial polarization is caused by the existence of multiple heterogeneous interfaces among NG/SnO_2_, NG/paraffin, SnO_2_/paraffin. In addition, the NG with residual defects as new polarization and scattering centers, resulting in a steady dielectric relaxation.

**Fig. 7:**
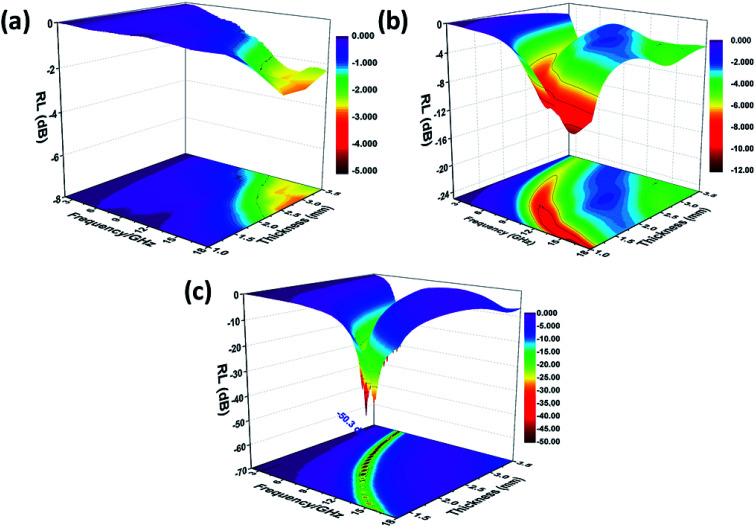
Three-dimensional RL values of SnO_2_ hollow spheres (a), NG (b) and NG/SnO_2_ hollow spheres hybrids (c).

Based on the generalized transmission line theory, the reflection loss (RL) curve of the absorber can be calculated based on the complex permittivity and permeability for the given absorber thickness and frequency by the following equation:^56^2
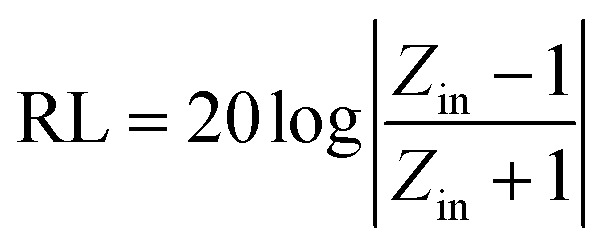
3
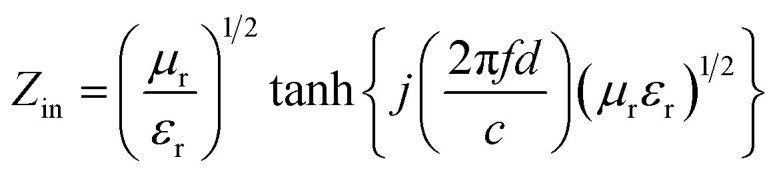
where *Z*_in_ is the input impedance of absorber, *f* is the frequency of the electromagnetic wave, *d* is the thickness of a microwave absorber, *c* is the velocity of light in vacuum. When the RL is below −10 dB and −20 dB, more than 90% and 99% electromagnetic energy is absorbed, and the bandwidth below −10 dB is often defined as the effective absorption bandwidth. Fig. 7(a) shows the calculated RL of SnO_2_ hollow spheres, it can be seen that the SnO_2_ hollow spheres exhibits poor microwave absorption properties within 2–18 GHz, the minimum RL value is only −2.8 dB at 17 GHz with the thickness of 2.5 mm. The minimal RL for NG is −10.86 dB which is better than the RGO (−7.5 dB).^11^ Nevertheless, the effective bandwidth is narrow due to the poor impedance matching of NG. The RL of the NG/SnO_2_ hollow spheres hybrids with different thickness are shown in Fig. 7(c). It can be observed that the thickness of the absorber has a great influence on microwave absorption properties and the minimum RL shifts toward to lower frequency as the absorber thickness increases. For the NG/SnO_2_ hollow spheres hybrids, the RL values less than −10 dB and −20 dB are found in the wide frequency range of 4.5–18 GHz and 5–16.2 GHz range with a variation in absorber thickness from 1.3 mm to 3.5 mm, and a minimum RL of −50.3 dB is achieved at 8.6 GHz with the thickness of 2.3 mm. The effective absorption bandwidth (RL < −10 dB) is 4.2 GHz for NG/SnO_2_ hollow spheres hybrids with the thickness of only 1.3 mm, which is thin enough as an absorber.

**Fig. 8:**
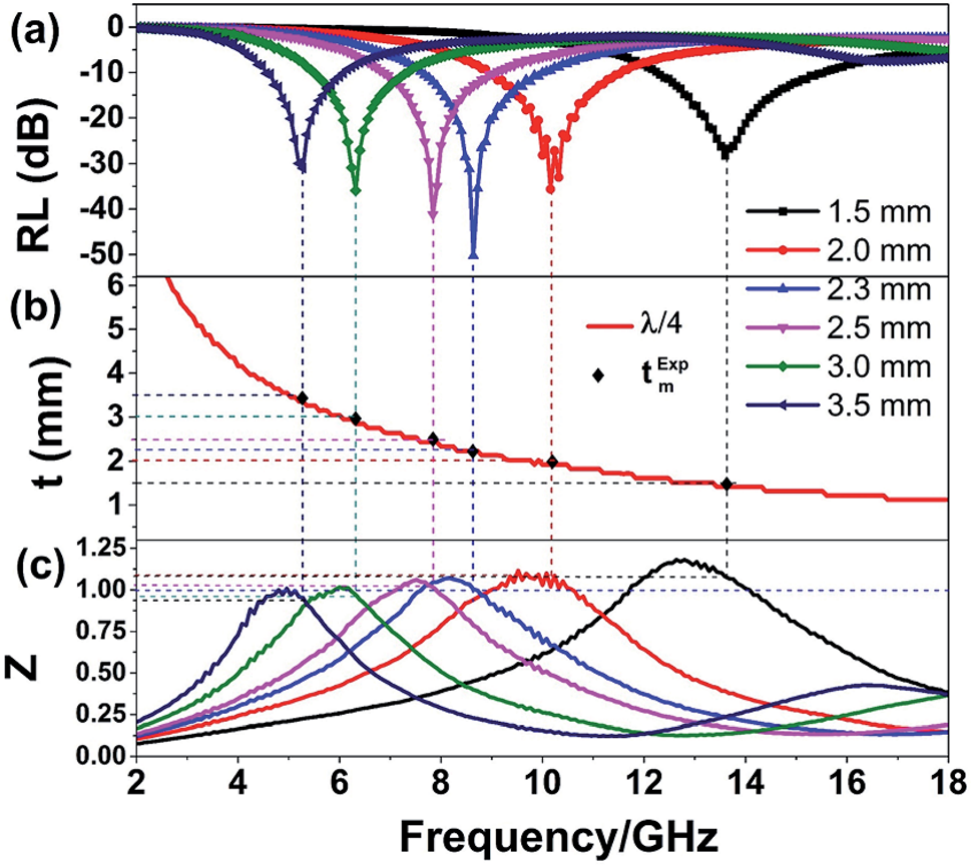
The RL values (a), the absorber thickness (*t*_m_) *versus* peak frequency (*f*_m_) under the quarter-wavelength cancellation model (b) and the modulus of normalized input impedance (c) for NG/SnO_2_ hollow spheres hybrids.

The quarter-wavelength cancellation model and zero reflection have been applied to analyze the enhanced microwave absorption. In the quarter-wavelength cancellation model, the relationship between the absorber thickness (*t*_m_) and the peak frequency (*f*_m_) can be described by the following equation:^1,6,57,58^4
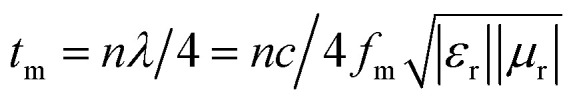
according to eqn (4), the EM wave reflected from the air–absorber interface and the absorber–metal interface are out of phase by 180°, resulting in an cancellation at the air–absorber interface.^1,6^ The minimum RL shifts toward the lower frequency as the absorber thickness increase also can be explained by the quarter-wavelength cancellation model. The black squares in Fig. 8(b) achieved from the RL curves directly in Fig. 8(a) are the matching thickness (donated as *t*^exp^_m_), almost locating on the *λ*/4 curve, which demonstrating that the good EM wave absorption properties of NG/SnO_2_ hollow spheres hybrids can be explained by the quarter-wavelength cancellation model. Apart from dielectric loss and magnetic loss, the RL is highly relevant to the impedance matching characteristic. When *Z* = |*Z*_in_/*Z*_0_| = |*ε*_r_/*μ*_r_|^1/2^|tanh{*j*(2π*fd*/*c*)(*ε*_r_*μ*_r_)^1/2^}| is close to 1, resulting in zero reflection at the air–absorber interface, most of EM wave can enter into the absorber. Fig. 8(c) shows the *Z*–*f* curves of the NG/SnO_2_ hollow spheres hybrids, in which the relationship between *f*_m_ and *Z* at the matching thickness is indicated by the dash lines. The minimum RL value can be obtained and the corresponding *Z* is close to 1 when the matching frequency is 8.6 GHz, and the corresponding matching thickness (2.3 mm) is almost on the *λ*/4 curve. Such high performance EM wave absorption properties of NG/SnO_2_ hollow spheres hybrids are ascribed to the combined contribution from quarter-wavelength interference cancellation and impedance matching characteristic.

**Fig. 9:**
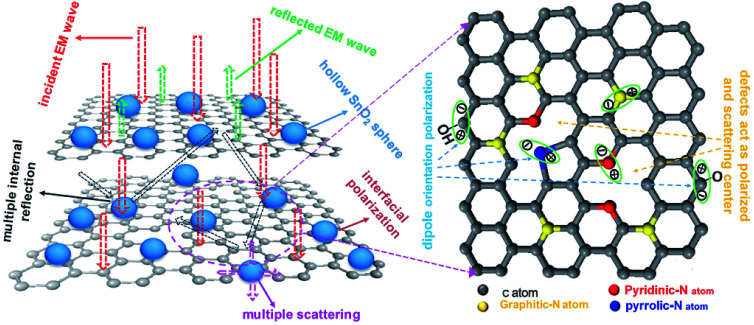
Possible mechanism of microwave absorption in NG/SnO_2_ hollow spheres hybrids.

Fig. 9 shows the schematic illustration of EM absorption mechanisms in NG/SnO_2_ hollow spheres hybrids. The excellent microwave absorption properties of NG/SnO_2_ hollow spheres hybrids may be explained by the following facts. First, the residual defects and groups in NG which can act as polarized and scattering center introduce defect polarization relaxation and multiple scattering. Second, the introduction of SnO_2_ hollow spheres hybrids in NG plays an important role in increasing the microwave absorption properties, which not only bring into better impedance matching, but also strengthen interfacial polarization. Third, the hollow SnO_2_ spheres anchored on the RGO sheets can provide more sites for scattering of electromagnetic wave. Forth, there are multiple internal reflection between NG which produce further dissipate more electromagnetic energy. From the above discussion, it can be concluded that the enhanced microwave absorption performance of NG/SnO_2_ hollow spheres hybrids is ascribed to the dielectric loss, better impedance matching, multiple internal reflection and quarter-wavelength interference performance.

## 4. Conclusion

In summary, the NG/SnO_2_ hollow spheres hybrids, in which SnO_2_ hollow spheres with the average diameter of 220 nm anchored on NG sheet uniformly were synthesized. The NG/SnO_2_ hollow spheres hybrids exhibit a maximum RL value of −50.3 dB at 8.6 GHz with a matching thickness of only 2.3 mm. The effective absorption bandwidth (RL < −10 dB) is 4.2 GHz for NG/SnO_2_ hollow spheres hybrids with the thickness of only 1.3 mm. The enhancement of microwave absorption for NG/SnO_2_ hollow spheres hybrids is attributed to the synergistic effect of dielectric loss, better impedance matching, multiple internal reflection and quarter-wavelength interference performance. These results indicate that the NG/SnO_2_ hollow spheres hybrids are ideal candidate as microwave absorbing materials.

## Conflicts of interest

There are no conflicts of interest to declare.

## Supplementary Material
